# Cystic echinococcosis in cattle slaughtered at a slaughterhouse in Gessa, southern Ethiopia

**DOI:** 10.1016/j.parepi.2022.e00262

**Published:** 2022-07-09

**Authors:** Mesfin Mathewos, Deneke Dawa, Metages Yirgalem, Tesfaye Denano, Haben Fesseha

**Affiliations:** a School of Veterinary Medicine, Wolaita Sodo University, Wolaita Sodo, Ethiopia; b Loma Bossa Woreda livestock and fishery resource office, Dawuro zone, Ethiopia; c College of Veterinary Medicine, Haramaya University, P.O. Box 138, Dire Dawa, Ethiopia; d College of natural and computational science, Wolaita Sodo University, Wolaita Sodo, Ethiopia

**Keywords:** Abattoir, Cystic echinococcosis, Ethiopia, Gessa, Zoonosis

## Abstract

Cystic echinococcosis is caused by the metacestode of the zoonotic flatworm *Echinococcus granulosus*. A cross-sectional study was conducted from October 2020 to August 2021 at the Gessa municipality abattoir in southern Ethiopia, through regular meat examinations and cyst characterization to determine the prevalence, organ distribution, and fertility of Cystic echinococcosis. The overall prevalence of *Echinococcus granulosus sensu lato* was 17.9% (69/384) and has shown a statistically significant association (*p* < 0.05) with the body condition of cattle but not with other putative risk factors (*p* > 0.05). The odds of poor body condition cattle infected with CE was 1.29 times higher than medium body condition (OR = 0.506; CI = 0.566–2.96). Based on organ distribution of cysts, the lungs and liver were the most affected organs having a statistically significant difference (*p* < 0.05) with the prevalence of CE. Based on the size of cysts, small (47.9%) size cysts revealed the highest prevalence as compared with large (33.3%) and medium (18.8%) sized cysts. On cyst characterization, the majority of Cystic echinococcosis were sterile cysts (55.4%) followed by fertile (38.8%) and calcified (8.7%) cysts and revealed a statistically significant difference (*p* < 0.05). Cyst number, organ localization, fertility of cyst, and cyst size have shown a strong positive correlation (*r* = 0.8686, *r* = 0.8393, *r* = 0.9506, and *r* = 0.9189) with the prevalence of CE, respectively. The presence of an overall high prevalence of *Echinococcus granulosus sensu lato* in the present study required urgent action to interrupt the life cycle of Cystic echinococcosis and further studies using molecular techniques to characterize the circulating genotypes to prevent the risk of zoonosis in humans in the study area.

## 1 Introduction

Ethiopia is considered to have the largest livestock population in Africa. It is estimated that the country hosts approximately 54 million cattle, 25.5 million sheep, 24.06 million goats, 50.38 million poultry, 1 million camels, and 5.21 million beehives (Asegede et al., 2015). Livestock production in Ethiopia is a fundamental part of almost every agricultural system in the highlands and is a major occupation in the lowlands (Hassen et al., 2007). Despite the large population, livestock productivity in Ethiopia remains low due to high livestock disease (parasitosis) (Guduro and Desta, 2019), malnutrition, and management constraints (Kumsa, 2019).

Cystic echinococcosis (CE), also known as hydatid disease, is a zoonotic disease with a worldwide distribution (Corrêa et al., 2018) including Ethiopia (Kumsa, 2019). It is caused by the metacestode of the tapeworm *Echinococcus granulosus (*Al-Khalidi et al., *2020**)* which can infect a variety of intermediate hosts, including livestock, such as sheep, goats, cattle, camels, buffaloes and pigs, and also humans (Corrêa et al., 2018; Monteiro et al., 2016). The CE of *Echinococcus granulosus sensu lato* develops as unilocular fluid-filled bladders within the infected internal organs (mainly liver and lungs, spleen, heart, and kidneys) of the intermediate hosts (Corrêa et al., 2018; Singh et al., 2014). As a result, CE inflicts enormous economic damage on domestic ruminants due to the condemnation of the affected organs and the lowering of meat, milk, and wool production in slaughtered animals in Ethiopia (Melaku et al., 2012).

Cystic echinococcosis in cattle has been conveyed from some parts of the country (Abebe et al., 2014; Mersie, 1993; Negash et al., 2013; Kumsa, 1994). Thus, wide-ranging and up-to-date evidence is anticipated for the effective control and prevention of CE in the country (Jobre et al., 1996; Magambo et al., 2006).

At the abattoir, detecting CE during routine meat inspection was led to condemnation of the infested offal (mainly livers and lungs). Fertile cysts (with viable protoscoleces) in livestock offal are very important for the continuation of the transmission cycle (Torgerson et al., 2003). The economic cost of CE in livestock can be divided into direct costs (mainly the loss of revenue through the condemnation of offal) and indirect costs (reduction in growth, fecundity, and milk production of infected animals) (Abdulhameed et al., 2018). Despite its great economic and health importance, studies regarding the prevalence of *E. granulosus* are few in the Gessa municipality abattoir in southern Ethiopia and do not provide sufficient information on the geographical presence and aetiological agents of CE. Therefore, to explore the prevalence and associated risk factors, organ distribution, and fertility of CE in cattle slaughtered at the Gessa municipality abattoir in Ethiopia.

## 2 Materials and methods

### 2.1 Study area

The study was conducted at the Gessa municipality abattoir in the Loma bossa district of the Dawuro zone, southern Ethiopia. It is surrounded by the Gamogofa Zone to the south, Esera to the west, Maleka to the northwest, Zaba Gazo Woreda to the north, and the Wolaita Zone to the east. The geographical location of the Loma bossa district is between 6°42′13″-6°53′48″N lat. and 37°00′20″E-37°15′48″E long. The elevation of the district ranges from 700 m to 2600 m above sea level. The average temperature is 22 °C, and the minimum and maximum temperatures are 15 °C and 29 °C, respectively. The main rainy season of the Woreda is June to September (the long rainy season), the short rainy season is March to April, and the dry season is October to May. The average annual rainfall is 1046 mm, ranging from 780 to 1312 mm. The Woredas economy is primarily based on mixed agriculture. The livestock population in the study area is estimated to be 347,990 cattle, 9767 sheep, 97,387 goats, 4860 horses, 2759 mules, 1699 donkeys, 63,042 poultry, and 2717 beehives (Ambushe and Gebre, 2020; Wolka et al., 2013).

### 2.2 Study animals

The animals included in the study were both male and female crossbred and native cattle slaughtered at the Gessa abattoir. The age, sex, breed, production system, and origin of each animal were recorded during the abattoir survey and divided into two groups adults (7–9 years) and the elderly (10+) as stated by (Herenda et al., 1994). The age of each study cow was estimated based on the dentition formula described by (Pace and Wakeman, 2003). The body conditions were categorized as good, medium, and poor, as explained by Nicholson and Butter (Nicholson, 1986).

### 2.3 Study design

A cross-sectional study was conducted from October 2020 to August 2021 to determine the prevalence, organ distribution, and characteristics of CE in cattle slaughtered at the Gessa municipality abattoir in Loma bossa district of the Dawuro zone, southern Ethiopia.

### 2.4 Sampling method and sample size determination

A simple random sampling technique was used in the cave to select the required number of study animals. A total of 384 cattle was examined to determine the presence of CE by postmortem examination of different visceral organs. The sample size was determined using 50% expected prevalence, 5% desired absolute precision, and 95% confidence interval based on the formula indicated by Thrusfield (Thrusfield, 2018);

$$\frac{N=Z^{2*}\;\text{Pexp}\;\left(1-\text{Pexp}\right)}{d^{2}}$$

Where, n = required sample size,

Z = statistic for level of confidence,

P_exp_ = expected prevalence,

d = desired absolute precision.

### 2.5 Study method

#### 2.5.1 Ante mortem examination

Immediately before slaughter, a complete ante mortem examination survey of cattle was conducted. Cows were examined for obvious signs of illness at rest and/or in gesture. All cows were tagged with an identification number before slaughter, based on the markings listed on the body surface using ink when the cows entered the stables.

#### 2.5.2 Postmortem inspection

Postmortem inspection was performed according to procedures recommended by food and agricultural organizations (Herenda et al., 1994). The internal organs, especially the lungs, liver, heart, spleen, and kidneys, were carefully examined by visualization, palpation, and incision. Organs containing cysts from infected animals were collected and multiple cysts were counted and recorded for each organ. The size of the diameter of the collected cysts was measured and classified into small (<5 cm diameter), medium (5 cm to 10 cm diameter), and large (>10 cm diameter) as described by (Abebe et al., 2014; Negash et al., 2013; Kumsa, 1994). The fertility of each cyst was determined after reducing the pressure of the cyst fluid using a sterile hypodermic needle. The cyst was then incised with a sterile scalpel blade and the contents were poured into a glass Petri dish for inspection. The presence of protoscolices, which appear as white spots on the embryonic epithelium and attach to the germ layer in the form of a brood capsule or cystic fluid, was considered an indicator of fertility. Sterile cysts are characterized by their smooth inner layer, and the fluid they contain is usually slightly cloudy, but cysts identified as calcified make a gritty sound when incised. Fertile cysts were subjected to viability tests. Droplets of sediment containing protoscolices were placed on a microscope slide, covered with a cover glass, and an amoeba-like peristaltic motion was observed with a 40× objective lens. For clear visibility, a drop of 0.1% aqueous eosin solution was added to an equal volume of precipitate containing protoscolices in the hydatid fluid on the microscopic slide (Negash et al., 2013). This technique distinguishes between dead (red) protoscolices and lives (colorless) protoscolices.

### 2.6 Data analysis

Data obtained from ant-mortem (origin, sex, age, breed, and body conditions) and post-mortem findings were coded and uploaded to Microsoft excel 2003 spreadsheet computer program. The data were analyzed by using STATA for windows version 20 and a chi-square test and multiple logistic regression analysis was applied to compare the prevalence of CE among cattle of different sex, age, breed, origin, and production system. A statistically significant association between variables is considered to exist if the *p*-value is <0.05 at a 95% confidence interval.

### 2.7 Ethics approval and consent to participate

The Wolaita Sodo University of Research Ethics and Review Committee provided ethical approval for this research. Before collecting samples, verbal consent was also sought from cattle owners to take samples from their cattle following strict hygienic measures. The best practice guidelines for animal care were followed as to the purpose of the study, and the Wolaita Sodo University of Research Ethics and Review Committee approved the verbally informed consent process.

## 3 Results

### 3.1 Prevalence of CE and associated risk factors

Out of 384 cattle examined, 69 (17.9%) had cysts and had one or more cysts in various internal organs (lungs, liver, spleen, kidneys). Among the putative risk factors, the prevalence of CE showed a statistically significant variation (*p* = 0.009) in the body condition of cattle. However, other factors did not show a statistically significant difference (*p* > 0.05) in the existence of CE. The odds of poor body condition cattle infected with CE was 1.29 times higher than medium body condition (OR = 0.506; CI = 0.566–2.96) when cattle with good body condition was kept constant. Cattle which was originated from low land, managed in semi-intensive production system, local breed, and old aged were 0.672, 0.638, 0.752, 1.32, and 1.49 times infected by CE than their respective groups (Table 1).

**Table 1:** Prevalence of CE in cattle.

Risk factors		Number of examined	No of infected	Prevalence (%)	OR	X^2^	p-value	[95% Conf. Interval]
Origin	High land	289	56	19.4	Ref	1.5721	0.210	Ref 0.345–1.31
	Low land	95	13	13.6	0.672			
Production system	Extensive	282	55	19.5	Ref	1.6966	0.193	0.331–1.22 Ref
	Semi-intensive	102	14	13.7	0.638			
Breed	Local	349	62	17.78	0.752	0.1078	0.743	0.304–1.86 Ref
	Cross	35	7	20	Ref			
Sex	Male	274	52	18.9	1.32	0.6611	0.416	0.717–2.45 Ref
	Female	110	17	15.4	Ref			
Age	Adult	222	35	15.7	Ref	1.7326	0.188	Ref 0.872–2.56
	Old	162	34	20.9	1.49			
Body condition	Good	52	12	23.1	Ref	9.4895	0.009	Ref 0.566–2.96 0.239–1.07
	Medium	254	35	13.7	0.506			
	Poor	78	22	28.2	1.29			
Overall		384	69	17.9				

### 3.2 Cyst size in relation to involved organs

Cysts were differentiated into small, medium, or large size cysts based on their size in cattle. The study revealed that small (47.9%) size cysts have the highest proportion than those with large (33.3%) and medium (18.8%) size cysts (Table 2).

**Table 2:** Distribution of cysts in different organs based on their size in cattle.

Cyst type Freq(%)	Organ				X^2^	P-value
	Lungs	Liver	Spleen	Kidneys		
Large 23(33.3%)	11(34.3%)	3(14.3%)	5(50%)	4(80%)	384	0.0001
Medium 13(18.8%)	7(21.8%)	4(19.1)	0(0%)	2(20%)		
Small 33(47.9%)	14(43.7%)	14(66.6%)	5(50%)	0(%)		

### 3.3 CE distribution in different organs

The study showed that the lungs and liver were the most affected organs in cattle, with the spleen and kidneys having a prevalence rates of 8.3%, 5.4%, 2.6%, and 1.5%, respectively but no cyst was found in the heart. This study detected a total of 321 cysts from various organs with a prevalence of 48.3%, 33.3%, 15.6%, and 2.8% from the lungs, liver, kidneys, and spleen. Both fertile and non-fertile cysts were detected in cyst characterization tests, except for the kidney, where only sterile cysts were detected. Out of 125 (38.9%) fertile cysts, 18% (58/321) were viable and 20.8% (67/321) were non-viable cysts. The majority of bovine CE were sterile cysts (55.4%) (Table 3).

**Table 3:** Type of cysts and their numbers in different organs.

Organ	Number (%) of positive organs	Type of cysts and their numbers in different organs				Total cysts recovered	X^2^ (p-value)
		Fertile cysts (%)		Non-fertile cysts (%)			
		Viable	Non-viable	Sterile	Calcified		
Lungs	32(46.3)	38(24.5)	37(23.8)	76(49)	4(2.5)	155 (48.3)	384 (0.0001)
Liver	21(30.4)	10(9.3)	26(24.2)	52(48.5)	19(17.7)	107 (33.3)	
Spleen	10(14.5)	0	4(44.4)	5(55.5)	0	9 (2.8)	
Kidneys	6(8.7)	0	0	45(90)	5(10)	50 (15.6)	
Total	69(17.9)	58(18)	67(20.8)	178(55.4)	28(8.7)	321(100)	

### 3.4 Correlation analysis of CE

Cyst number, organ localization, fertility of cyst, and cyst size have shown a strong positive correlation (r = 0.8686, r = 0.8393, r = 0.9506, and *r* = 0.9189) with the prevalence of CE, respectively (Table 4).

**Table 4:** Correlation analysis.

	Hydatid cyst	Cyst number	Origin	Husbandry system	Breed	Sex	Age	Body condition	Organ localization	Fertility of cyst	Cyst size
Hydatid cyst	1.0000										
Cyst number	0.8686	1.0000									
Origin	−0.0640	−0.0370	1.0000								
Husbandry system	−0.0665	−0.0644	−0.0579	1.0000							
Breed	−0.0168	−0.0029	0.0348	0.0061	1.0000						
Sex	0.0415	0.0677	−0.0639	0.1463	−0.0005	1.0000					
Age	0.0672	0.0541	0.0235	−0.0839	−0.0226	−0.0186	1.0000				
Body condition	−0.1251	−0.1460	0.0504	−0.0462	−0.0827	−0.0250	0.0569	1.0000			
Organ localization	0.8393	0.7714	−0.0420	−0.0630	−0.0085	0.0627	0.0673	−0.0991	1.0000		
Fertility of cyst	0.9506	0.8370	−0.0532	−0.0277	−0.0145	0.0164	0.0346	−0.1189	0.7544	1.0000	
Cyst size	0.9189	0.7714	−0.0677	−0.0901	−0.0266	0.0530	0.0513	−0.1187	0.7789	0.8578	1.0000

## 4 Discussion

CE is known as a major disease in livestock and humans worldwide, and its incidence and economic importance have been reported in several studies from different regions of the world (Varcasia et al., 2020; Joanny et al., 2021; Budke et al., 2006; Agudelo Higuita et al., 2016; Craig et al., 2007). The overall prevalence of CE (17.9%) in cattle slaughtered at the Gessa municipality abattoir was consistent with the earlier reports of (Gebremeskel and Kalayou, 2009; Kebede et al., 2009a; Regassa et al., 2009; Njoroge et al., 2002) who reported a prevalence of 17.5%, 16%, and 15.4%, respectively. However, higher reports were previously reported by (Kumsa, 2019; Abebe et al., 2014; Negash et al., 2013; Jobre et al., 1996; Regassa et al., 2009; Kebede et al., 2009b; Kebede, 2010; Koskei, 1998; Kebede et al., 2009c; Berhe, 2009; Zewdu et al., 2010; Kumsa and Mohammedzein, 2014) who reported a prevalence ranging from 20.5%–84.3% from different parts of a country and across the globe by (Chihai et al., 2016; Moro and Schantz, 2006; Cardona and Carmena, 2013; Addy et al., 2012). On the other hand, a lower prevalence of CE has been previously reported by (Budke et al., 2006; Regassa et al., 2009; Poglayen et al., 2017; Omer et al., 2010). This variation in the prevalence of CE might be due to the differences in agroecology, the time of studies, stock density and migration of animals, livestock production systems, awareness, culture, and religion of society, and attitudes to dogs in different parts of the country (Abebe et al., 2014; Kumsa, 1994; Romig et al., 2011).

In this study, a statistically significant difference (*p* < 0.05) was seen between the body condition of cattle and the prevalence of CE. It was found that animals with poor body conditions had a large number of cysts counts which likely reflected the effects of cyst load in poor body conditioned animals. Polydorou (Polydorou, 1981) states that when the infection is moderate to severe, parasites can cause delayed performance and growth, poor meat and milk quality, and weight loss. In addition, there were no statistically significant differences in origin, breed, gender, or production system in the development of CE. The lack of significant differences in the prevalence of CE in cattle of different breeds, agroecology, and production systems might be due to a high population of stray dogs as well as wild dogs in close association with the family and farm animals in all agro-ecological zones of Ethiopia has been previously suggested by (Kumsa, 2019; Abebe et al., 2014; Negash et al., 2013). Furthermore, the outbreaks of CE in Ethiopia are due to the common practice of ruminants slaughtering in the backyard and roadside together with the widespread tradition of providing uncooked infected offal to dogs. In addition to these common practices, other factors support the spreading of CE including low public awareness. Moreover, lack of proper fences, disposal pits from abattoirs, lack of habit of disposing of dead wildlife or domestic animals, and unburied leftovers for carnivorous animals create favorable conditions for environmental contamination by *E. granulosus sl* eggs that maintain the life cycle of *E. granulosus* in stray dogs (Kebede et al., 2009a; Regassa et al., 2009; Zewdu et al., 2010).

As reported in Table 1, the prevalence of CE was higher in old animals (20.9%) compared to adults (15.7%). Similar reports were previously indicated by (Regassa et al., 2009; Zewdu et al., 2010) from Ethiopia and by (Azlaf and Dakkak, 2006; Adinehbeigi et al., 2013; Carmena and Cardona, 2013) from other countries. This may be primarily because older animals are exposed to *E. granulosus* eggs for longer periods, in addition to their weak immunity to fight against the infection (Cardona and Carmena, 2013; Himonas et al., 1987).

The finding of CE in lung and liver as compared to other organs has been previously reported by (Mersie, 1993; Kumsa, 1994; Jobre et al., 1996; Njoroge et al., 2002; Zewdu et al., 2010; Omer et al., 2010; Bekele and Butako, 2011; Kebede et al., 2011; Scala et al., 2017) in which the most affected organs of cattle were the lung (55.8%) and liver (71.2%), and only in one cattle, CE was found in the kidney with a prevalence of 1.9% (Scala et al., 2017) which was in line with current finding that revealed a high prevalence of CE in the lung (46.3%) and liver (30.4%) as compared with other organs. The increased frequency of infection in the lungs and liver could be since wandering CE oncospheres that enter the subepithelial capillaries of the intestine or the lacteal must first pass through the great capillary bed of the hepatic and pulmonary filtering systems before affecting any other peripheral organ (Kebede et al., 2009b; Kumsa and Mohammedzein, 2014). Because the lungs have the greatest capillary beds, oncospheres entering the vena cava with lymph will be filtered out and stuck in the lung first, forming a cyst in the process (Cardona and Carmena, 2013; Ibrahim, 2010; Taylor et al., 2007; Abebe et al., 2021).

Higher numbers of large, medium, and small sized cysts were found in lungs of cattle than in the liver. A similar finding was obtained by (Kumsa, 2019) in Addis Ababa abattoir enterprise; (Abunna et al., 2012) in Kombolcha ELFORA abattoir and (Asrat, 1996) in South Wollo. The reason for higher percentage of medium and large cysts in the lungs due to softer consistency of the lung while the higher yield of calcified cysts in liver could be attributed to relatively higher reticuloendothelial cells and abundant connective tissue reaction of the organ. The high proportion of small cysts may be due to immunological response of the host which might preclude expansion of cyst size (Abebe et al., 2014; Kumsa and Mohammedzein, 2014).

Examination of cyst fertility and survival revealed that approximately 55.4% were sterile, 38.8% were fertile, and 8.7% were calcified cysts. Similar reports were previously reported by (Abebe et al., 2014; Regassa et al., 2009; Getaw et al., 2010). The high prevalence of sterile cyst in the current study suggest that the majority of bovine CE are not infectious to the final host. This finding supports the previous discussion of several researchers who claimed that sheep play a greater role as an intermediate host for CE than cattle in Ethiopia (Kumsa and Mohammedzein, 2014; Erbeto et al., 2010). However, the high prevalence of fertile CE (38.9%) in cattle slaughtered at the Gessa municipality abattoir shows that cattle still have some potential source of infection to dogs and another final host of this parasite. This observation is consistent with previous results (Kumsa, 1994). The fertility of cysts could be affected by differences in the strain of *E. granulosus* (Njoroge et al., 2002; Romig et al., 2011). A correlation analysis of CE among cyst number, organ localization, fertility of cyst, and cyst size revealed a strong positive correlation. Cysts can have different fertility rates depending on geographic location, host, location, size, and cyst type (Ibrahim, 2010). In addition, the fertility of intermediate host cysts may also be genotype-dependent, but unfortunately, no genotype studies are available for any host in Ethiopia. In this study, the highest prevalence and fertility of pulmonary CE were observed due to practices of feeding uncooked lungs to dogs than other organs may play a major role in the transmission of *E. granulsous sensu lato (either E. granulous sensu stricto or E. ortleppi)* in Ethiopia.

## 5 Conclusion

The results reported here indicate that CE is widespread in cattle slaughtered at slaughterhouses in the city of Gessa. Observation of fertile cysts in the investigated organs still plays a role in the life cycle of this serious zoonotic disease in cattle and is a potential risk of transmission to other intermediate hosts and the population of the study area. Therefore, safe disposal of affected internal organs and education of the population about CE in the area are needed to interrupt the life cycle of CE. In addition to that further studies using molecular techniques should be conducted to characterize the circulating genotypes to prevent the risk of zoonosis in humans in the study area.

## Ethics approval and consent to participate

The Wolaita Sodo University of Research Ethics and Review Committee provided ethical approval for this research. Before collecting samples, verbal consent was also sought from cattle owners to take samples from their cattle following strict hygienic measures. The best practice guidelines for animal care were followed as to the purpose of the study, and the Wolaita Sodo University of Research Ethics and Review Committee approved the verbally informed consent process.

## Funding

This work was not supported by any funding source or institution.

## Data availability statement

The data will be provided upon the request of the corresponding author.

## Consent for publication

Not applicable.

## Availability of data and materials

The datasets used and analyzed during the current study are available from the corresponding author on reasonable request.

## Authors' contributions

MM have made substantial contributions to conception and design, or acquisition of data, manuscript write-up and interpretation of data. DD has been involved in sample collection and writing and revising the manuscript. MY have been involved in recruiting the manuscript or revising it critically for important intellectual content. TD have been participated in the design of the study and performed statistical analysis. HF have been involved in recruiting the manuscript or revising it critically for important intellectual content. All authors read and approved the final manuscript.

## Authors' information

Not applicable.

## Competing interests

All authors have seen and approved the final version of the manuscript being submitted. They warrant that the article is the authors' original work, has not received prior publication and is not under consideration for publication elsewhere.
